# Impact of Obstructive Sleep Apnea Treatment on Marital Relationships: Sleeping Together Again?

**DOI:** 10.7759/cureus.46513

**Published:** 2023-10-05

**Authors:** Catarina Cascais Costa, Vera Afreixo, João Cravo

**Affiliations:** 1 Department of Pulmonology, Centro Hospitalar do Baixo Vouga, Aveiro, PRT; 2 Center for Research and Development in Mathematics and Applications, Department of Mathematics, University of Aveiro, Aveiro, PRT

**Keywords:** relationship, sleep apnea treatment, sleep apnea, sleep, couples

## Abstract

Objective

We assess the impact of obstructive sleep apnea (OSA) treatment on the quality of marital relationships. Moreover, we evaluate the proportion of couples sleeping separately before treatment and whether there was any change after treatment began, which is still little explored in the literature.

Methods

A prospective study was conducted between April 2021 and April 2023, with users diagnosed with OSA in a level 2 hospital in Portugal. A questionnaire was applied before and after the start of treatment to both user and partner, which included questions on whether they slept together or separately, the disturbing factors of sleep quality, and satisfaction with the marital relationship. Statistical analysis was performed using R (version 4.2.2; R Development Core Team, Vienna, Austria).

Results

Seventy questionnaires were applied, 79% to male users. Forty-one percent of users reported that they slept at least once or twice a month separated from their spouse, and, of these, 41% always slept in separate rooms. The chief complaints of partners not sleeping together were snoring (86%), restless sleep (17%), and witnessed apnea (14%). After treatment, 72.4% started to sleep together again, with a statistically significant difference in the condition before and after intervention. Among all patients, 69% said that their personal lives had improved and, when asked the same question to their spouse, 74% recognized the benefit of therapy.

Conclusion

Starting treatment positively influenced the quality of the marital relationship of users and their partners, with a statistically significant proportion of couples sleeping together again.

## Introduction

Obstructive sleep apnea syndrome (OSA) is highly prevalent in our population and worldwide, affecting an estimated 10%-17% of adults [1,2]. Typical symptoms include daytime sleepiness, increased cardiovascular disease, depressive symptoms, and decreased quality of life [3-5]. Nocturnal symptoms such as snoring, witnessed apnea, and frequent awakenings affect not only the patient's sleep quality but also that of their partners [3,5-7], namely, due to concern over witnessed apnea [5,8]. All these symptoms can contribute to changes in the dynamics of relationships between couples and may even lead to one of the partners leaving the bedroom [7,9-11].

The preferred treatment for OSA is based on the application of positive airway pressure [1,11]. Commencing the recommended treatment for OSA has a positive impact on patient quality of life, with emphasis on physical health, increased vitality, and improvement in mental health and social relationships [2,8,10,12].

Despite all the positive effects described, the rate of nonadherence to positive pressure treatment ranges from 46% to 83% [3], which is often associated with higher morbidity, mortality, and economic costs [2]. Conflict between couples may contribute to reduced adherence to treatment [12]. Support from partners, friends, and family is a predictor of adherence, so marital status should be considered when approaching treatment [1]. Thus, providing information to the partner about the disease and treatment can help increase adherence [11,13].

Data indicate that, for the partner, the use of positive airway pressure may also be associated with discomfort in the quality of sleep, namely, because of the noise it can produce. Nonetheless, the balance is positive because of the proven improvement in the patient's sleep quality [10]. This benefit may lessen conflict between spouses [6], which in turn may lead to couples sleeping together again. However, evidence remains inconsistent regarding the benefits for couples [5].

The authors conducted a study to investigate the impact of the introduction of therapy for OSA on the quality of marital relationships. They also assessed the proportion of couples who slept separately and the effect of starting treatment on this specific and still little-studied aspect of relationships.

## Materials and methods

The authors conducted a prospective study between April 2021 and April 2023 using a convenience sample of patients diagnosed with OSA in a level 2 hospital in Portugal.

Upon obtaining written informed consent, a questionnaire was applied before and after the beginning of treatment (Appendix 1), which included questions about the age and sex of both user and partner, whether they slept together or separately, the disturbing factors of sleep quality, whether they considered that sleeping separately affected their relationship, and if they would like to sleep together again. We also applied the relationship assessment scale [14], validated in Portugal [15] before and after starting treatment. Incompletely answered questionnaires were excluded from the analysis.

Descriptive analysis of the quantitative variables under study was performed by obtaining mean and standard deviation, and the qualitative variables by counting and percentages. To assess the effect of the intervention (beginning of treatment) on binary qualitative variables, McNemar’s test was used. Univariate logistic regression was used to analyze the association with the variable that records the occurrence of improvement in personal life after starting treatment.

Subsequently, a separate analysis was made of users who previously slept separately, at least once or twice a month. Data on the overall satisfaction of the user's and partner's relationship were compared between patients who went back to sleeping together and those who continued to sleep separately.

A comparison of variables between groups was performed using Fisher's exact test and described using frequencies and counts for qualitative variables and median and interquartile range for continuous variables. A comparison was also made between the groups that never slept apart and those that sometimes/always slept apart with items from the relationship assessment scale using Wilcoxon’s test.

Statistical analysis was performed using R (version 4.2.2; R Development Core Team, Vienna, Austria).

The Ethics Committee of Centro Hospitalar Baixo Vouga, Aveiro, Portugal, approved the study (Ref. 44-04-2021).

## Results

A total of 70 questionnaires were obtained. Most respondents were male (79%) and married (97%), with severe OSA. The sociodemographic characteristics of the patient, including the severity of OSA and their partners, are represented in Table 1.

**Table 1:** Sample characterization.

Characteristics	N = 70^1^
Gender	
Female	15 (21%)
Male	55 (79%)
Age	58 (11)
Marital Status	
Married	67 (96%)
Divorced	1 (1.4%)
Single	2 (2.9%)
OSA severity	
Severe	33 (47%)
Moderate	23 (33%)
Mild	14 (20%)
Partner Age	57 (12)
Partner Gender	
Female	52 (74%)
Male	18 (26%)
^1^n (%); Mean (SD)	

Of all respondents, 41% (n=29) slept apart from their spouse at least once a month, of which 21% (n=6) slept apart about one to two times a month, 38% (n=11) slept apart about one to two times a week, and 41% (n=12) always slept apart. In 31% (n=9) of cases, it was the patient who left the room; in 52% (n=19), it was the partner; and, in 17% (n=5), it was variable.

Of the patients who did not always sleep together, the partner's main complaint was snoring (86%), followed by restless sleep (17%), and witnessed apneas (14%).

On a scale of 1-5, patients who slept separately considered that doing so affected the relationship with a median of 3 (range between a minimum of 1 and maximum of 4) and would like to sleep together again at a median of 5 (range between a minimum of 4 and maximum of 5).

Commencing treatment led to a change in the condition of sleeping apart with statistically significant differences (McNemar's chi-square test=19.048, df=1, P value=1.275e-05). Of the 41% (n=29) of users who slept separately, 72.4% (n=21) began to sleep together. In no case did the initiation of treatment lead to patients starting to sleep separately.

After starting treatment, of the 70 patients questioned, 69% (N=48) considered that their personal life had improved and 74% (N=52) of partners also answered affirmatively to this question. This benefit reported by either patients or partners was not influenced by gender, age, OSA severity, reported symptoms, or the person who left the room.

Among couples who slept separately, in those who started sleeping together again, both patients and their partners reported greater improvement in their personal lives (38% versus 81% and 25% versus 95%, respectively), as demonstrated in Table 2.

**Table 2:** Comparison between the group of couples who started sleeping together again and the group who kept sleeping separately.

Characteristics	N	Overall, N = 29^1^	Kept sleeping separately N = 8^1^	Slept together again N = 21^1^	P value^2^
Patient-reported improvement in their personal life	29	20 (69%)	3 (38%)	17 (81%)	0.067
Partner reported improvement in their personal life	29	22 (76%)	2 (25%)	20 (95%)	<0.001
^1^n (%)					
^2^Fisher's exact test					

In the relationship assessment scale, there were no statistically significant differences between before and after treatment (Table 3).

**Table 3:** Relationship assessment scale results before and after starting treatment.

	Before treatment		After starting treatment	
1. How well does your partner meet your needs?	1	1 (1.4%)	2	1 (1.4%)
	2	1 (1.4%)	3	7 (10%)
	3	7 (10%)	4	25 (35.7%)
	4	25 (35.7%)	5	36 (51.4%)
	5	36 (51.4%)	Unknown	1
2. In general, how satisfied are you with your relationship?	1	0	1	0
	2	0	2	0
	3	7 (10%)	3	6 (8.6%)
	4	21 (30%)	4	20 (28.5%)
	5	42 (60%)	5	44 (62.9%)
3. How good is your relationship compared to most?	1	0	1	0
	2	0	2	1 (1.4%)
	3	8 (11.4%)	3	4 (5.7%)
	4	28 (40%)	4	38 (54%)
	5	34 (48.6%)	5	27 (38.6%)
4. How often do you wish you had not got into this relationship?	1	57 (81.4%)	1	58 (82.9%)
	2	10 (14.3%)	2	9 (12.9%)
	3	2 (2.9%)	3	1 (1.4%)
	4	1 (1.4%)	4	1 (1.4%)
	5	0	5	1 (1.4%)
5. To what extent has your relationship met your original expectations?	1	0	1	1 (1.4%)
	2	1 (1.4%)	2	0
	3	9 (13%)	3	7 (10%)
	4	37 (53%)	4	38 (54%)
	5	23 (33%)	5	24 (34%)
6. How much do you love your partner?	1	0	1	1 (1.4%)
	2	0	2	0
	3	6 (8.6%)	3	7 (10%)
	4	25 (35.7%)	4	13 (18.6%)
	5	39 (55.7%)	5	49 (70%)
7. How many problems are there in your relationship?	1	38 (54.3%)	1	45 (64.3%)
	2	21 (30%)	2	21 (30%)
	3	6 (8.6%)	3	2 (2.9%)
	4	4 (5.7%)	4	2 (2.9%)
	5	1 (1.4%)	5	0

The baseline level of satisfaction with the relationship (points 1-7, individually) of each patient did neither influence whether they slept together nor the improvement in the relationship after starting treatment.

## Discussion

In this study, the authors confirmed that the symptoms that most bothered partners were snoring, restless sleep, and witnessed apnea, which is in line with the literature [5-7].

The authors point out that in a significant percentage of the sample (41%), these complaints led one of the partners to leave the bedroom, damaging the relationship [7, 9-11], with the majority considering that sleeping separately affected the quality of their marital relationship and that they would like to sleep together again. This is a little-studied aspect, but it should be pivotal in assessing the quality of the relationship and adherence to treatment.

Upon starting treatment, 72.4% of couples who slept separately went back to sleeping together, which is an important marker of therapeutic success. The authors consider that, within the general assessment of the impact on the quality of life of sleep apnea, the specific impact on the marital relationship should be questioned and addressed.

Patients who have good personal relationships, namely, marital ones, are more likely to be successful in therapy [1,2,11,12]. On the other hand, this study reinforces the positive impact that the treatment itself has on the quality of the relationship, with 69% of patients and 74% of partners surveyed noticing these improvements. Thus, the authors consider that this issue should be used as a motivating agent for adherence to therapy.

The authors acknowledge the small sample size as a relevant limitation of this study, emphasizing that the period of data collection covered the COVID-19 pandemic, which had an incredibly significant impact on medical practice. Specifically, the institution of mandatory non-face-to-face consultations in Portugal encumbered the recruitment of patients for this study. Nevertheless, the authors consider the results obtained to justify their disclosure, since they explore a little-explored facet with significant relevance in the treatment of patients with OSA.

The authors also mention as a limitation to this study the nonspecific nature of the scale used to assess the quality of the relationship, which may make it difficult to perceive differences at the beginning of treatment. The authors highlight the importance of developing objective tools for evaluating with greater specificity the therapeutic intervention and its impact on the quality of the marital relations.

Among couples who slept separately, due to symptoms associated with sleep apnea, there was a statistically significant difference after starting treatment, with 72.4% sleeping together again.

## Conclusions

OSA is a prevalent pathology, with a very important economic and social impact. In this article, the authors propose to address the impact of the pathology as well as its treatment, namely, on the specific interference in the marital relationship. We found that the introduction of the treatment improved the quality of the relationship both from the perspective of the user and from the spouse, with a statistically significant proportion of couples sleeping together again.

This is an aspect that has not yet been fully explored and that should be addressed, as a parameter of quality of life and a motivator for adherence to treatment.

## Figures and Tables

**Figure 1:**
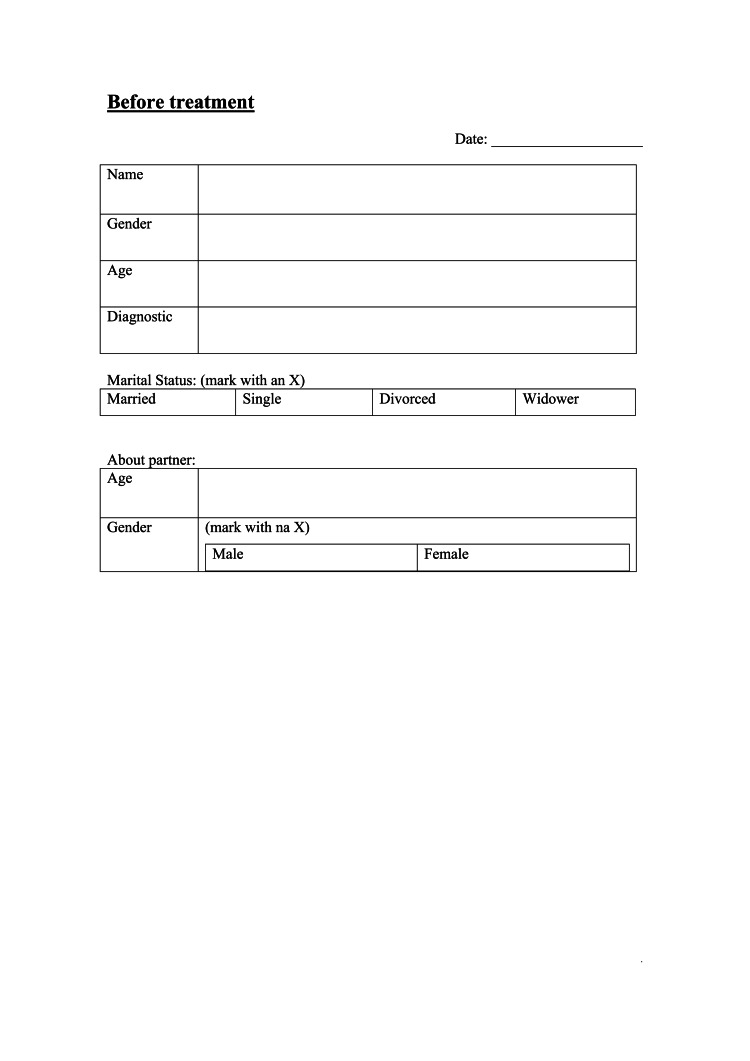
Questionnaire - Part 1

**Figure 2:**
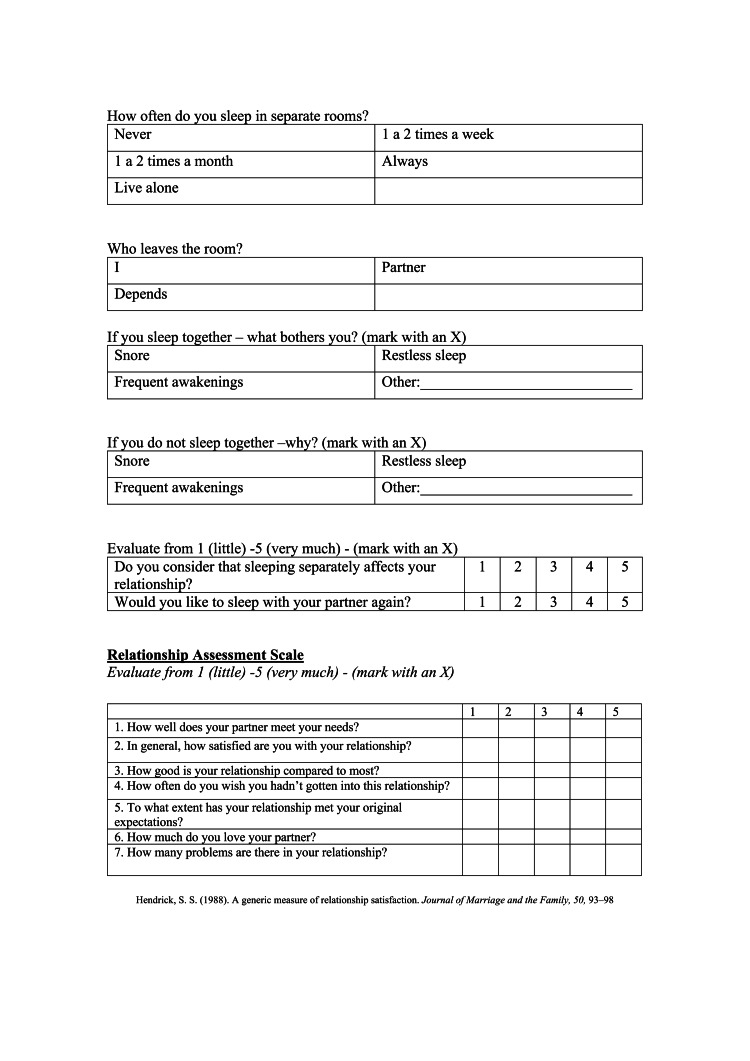
Questionnaire - Part 1.1

**Figure 3:**
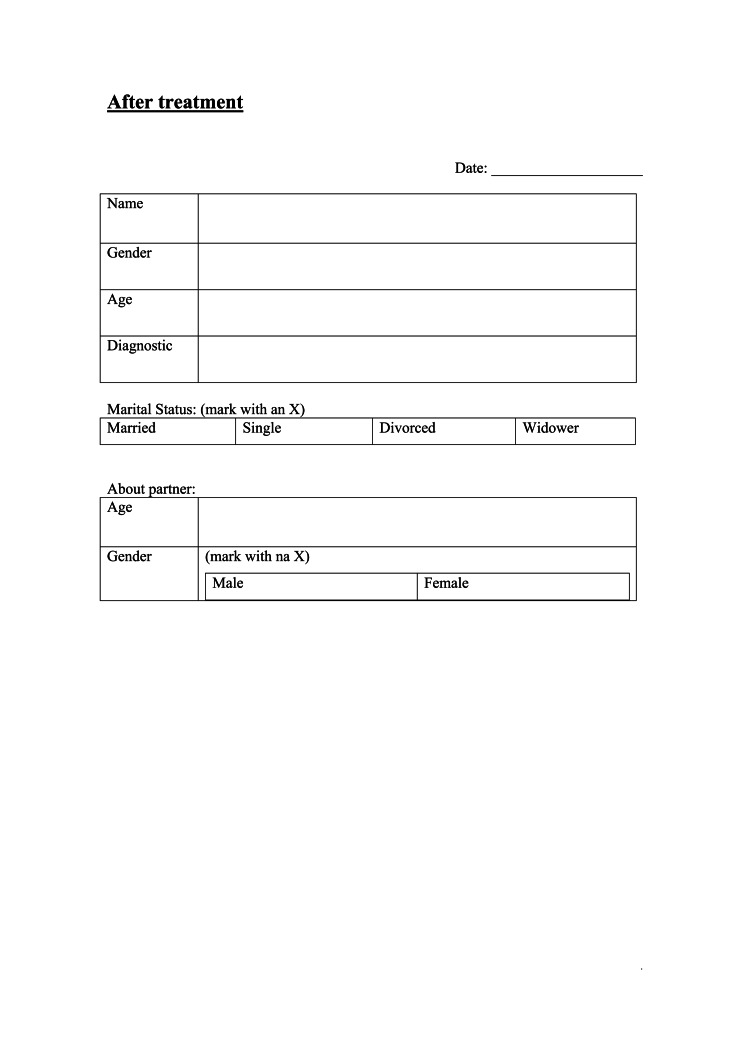
Questionnaire - Part 2

**Figure 4:**
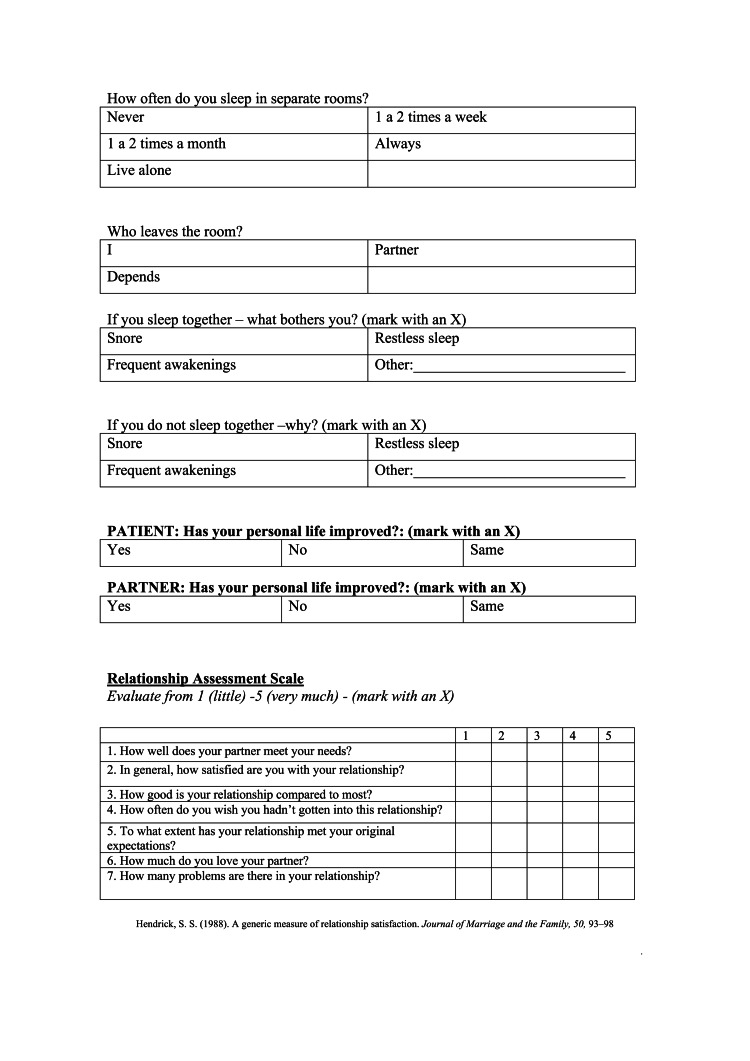
Questionnaire - Part 2.1
